# Role of the Microenvironment in Ovarian Cancer Stem Cell Maintenance

**DOI:** 10.1155/2013/630782

**Published:** 2012-12-24

**Authors:** Jennifer Pasquier, Arash Rafii

**Affiliations:** ^1^Stem Cell and Microenvironment Laboratory, Department of Genetic Medicine and Obstetrics and Gynecology, Weill Cornell Medical College in Qatar, Education City, Qatar Foundation, P.O. Box, Doha 24144, Qatar; ^2^Department of Genetic Medicine, Weill Cornell Medical College, New York, NY 10021, USA

## Abstract

Despite recent progresses in cancer therapy and increased knowledge in cancer biology, ovarian cancer remains a challenging condition. Among the latest concepts developed in cancer biology, cancer stem cells and the role of microenvironment in tumor progression seem to be related. Indeed, cancer stem cells have been described in several solid tumors including ovarian cancers. These particular cells have the ability to self-renew and reconstitute a heterogeneous tumor. They are characterized by specific surface markers and display resistance to therapeutic regimens. During development, specific molecular cues from the tumor microenvironment can play a role in maintaining and expanding stemness of cancer cells. The tumor stroma contains several compartments: cellular component, cytokine network, and extracellular matrix. These different compartments interact to form a permissive niche for the cancer stem cells. Understanding the molecular cues underlying this crosstalk will allow the design of new therapeutic regimens targeting the niche. In this paper, we will discuss the mechanisms implicated in the interaction between ovarian cancer stem cells and their microenvironment.

## 1. Introduction

Ovarian cancer remains a challenging condition for both clinicians and scientists. Indeed, it often presents as an advanced metastatic disease; however most patients are treated with a combination of major debulking surgeries and chemotherapy to achieve complete cytoreduction (no tumor residue) [1]. The clinical course of patients with no residue at the end of the treatment remains unpredictable with a group of early recurrence (refractory patients) [2]. The clinical trials of targeted therapies (trastuzumab, imatinib, etc.) as well as dose intensifications or use of several agents have failed to significantly improve outcomes [3–6]. Finally, procedures such as intraperitoneal chemotherapy or hyperthermic intraoperative chemotherapy have only a slight effect on prognosis with significant increase in overall morbidity [7].

The biology of ovarian cancers also has striking features; over the last decade the heterogeneity of ovarian cancers among and within subtypes has been illustrated by transcriptomic and genetic profiling [8]. Many authors have presented prognosis signatures without a clear translation to the clinical setting [9]. Recently, a broad study by The Cancer Genome Atlas (TCGA) has demonstrated among other findings that serous ovarian adenocarcinoma could be clustered in 4 different subtypes without being able to relay them to prognosis [10]. The mutational spectrum of ovarian cancers seems to be limited with most genetic events happening at the copy number variation level. Metastatic lesions have a genetic profile different to primary lesions, again reflecting tumor heterogeneity [11]. However the specific biological features responsible for recurrences have not been clearly identified. 

Recently, the concept of cancer stem cells (CSCs) has emerged as an alternative to the clonal theory of tumor evolution. Indeed among the heterogeneous populations constituting a tumor, a small proportion of cells (0.01% to 0.1%) have properties that mimic to certain extent normal stem cell biology: (i) self-renewal with asymmetric and symmetric cell division; (ii) recapitulation of the tumor heterogeneity in immune-suppressed mice; (iii) ability to undergo serial passages *in vitro* and *in vivo* due to unlimited division potential [12]. The role and biology of ovarian cancer stem cells have been already illustrated in other comprehensive reviews [13, 14]. The tumor is now perceived as a complex structure where the tumor cells closely interact with the stroma, which provides protumoral and prometastatic cues [15]. Our group has demonstrated the role of mesenchymal stem cells in transferring multidrug resistance protein (MDR) or inducing a prometastatic phenotype of ovarian cancer cells [16, 17]. Thus, microenvironment might have a real role in the biology of ovarian cancer stem cells (OCSCs). 

Here, we review the data about ovarian cancer stem cells and their interaction with the tumoral microenvironment. Understanding the molecular cues responsible for the crosstalk between the tumor and its stroma might help us design new therapeutic strategies aiming at disrupting specific prostemness tumor-stroma interaction rather than targeting tumor cells alone.

## 2. Ovarian Cancer Stem Cells

Genetic changes in regular stem cells might give rise to OCSCs [18, 19]. As the exact origin of ovarian cancer is still debated (ovarian surface epithelium versus fallopian tube) and its complexity is not limited to one subtype, characterization and definition of OCSCs have been really challenging. Besides, OCSCs can display different states (quiescent or proliferative) depending on the microenvironment and the cellular stresses such as chemotherapy which makes it more difficult to gather a unique definition [20, 21]. Currently surface markers or a particular phenotype (side population) are used to identify OCSCs.

**Figure 1:**
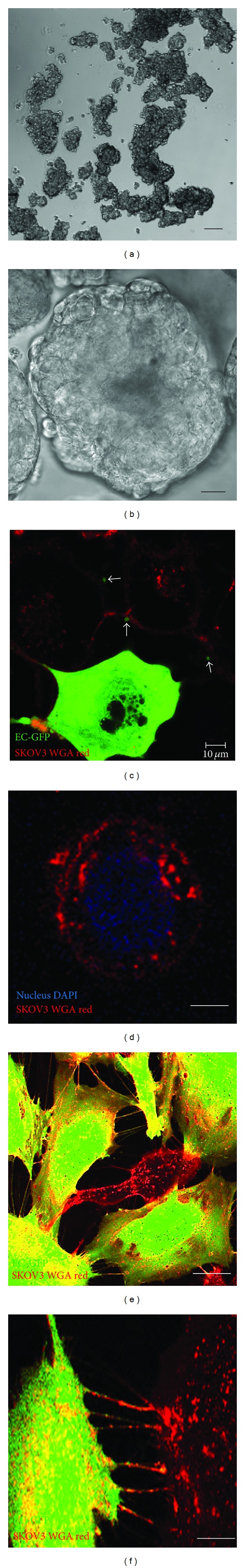
Ovarian cancer cells. (a) Ovarian cancer cell lines, SKOV3 in spheroid culture. Scale bar 100 *μ*m. (b) Confocal imaging of an SKOV3 sphere. Scale bar 20 *μ*m. (c) Coculture of endothelial cells- (ECs-) GFP (green) and SKOV3 (red). ECs secrete microparticles (arrows) which are uptaken by ovarian cancer cells. Scale bar 10 *μ*m. (d) Microparticles from ECs were tagged with WGA-alexa fluor 594 and added to culture of SKOV3 during 6 hours. Ovarian cancer cells are able to uptake ECsmicroparticles. Scale bar 5 *μ*m. (e)-(f) Coculture of ECs-GFP (green) and SKOV3 (red). Both cell types are interconnected by tunneling nanotubes. Scale bar 10 *μ*m.

The most commonly described marker is CD133. Different authors showed that CD133^+^ from cell lines or primary xenografts had greater capacity to initiate tumors than CD133^−^ [22, 23]. OCSCs were more comprehensively characterized by the combination of CD133 and the stem cells marker aldehyde dehydrogenase (ALDH) [24, 25]. Finally previously described CSCs markers CD44 and CD117 were used to characterize OCSCs. Cancer stem cells have the increased ability to be grown in 3D anchorage-independent culture setup as spheres (Figures 1(a) and 1(b)). The formation of primary and/or secondary sphere is currently routinely used to enrich and/or quantify the stem cell population [26]. The other striking feature of OCSCs is their chemoresistance and thus their potential role in residual and recurrent disease even if this has not been yet clinically demonstrated [22, 27, 28]. Indeed in ovarian cancer, CD44^+^CD117^+^ spheroids were resistant to chemotherapy and were able to initiate and propagate tumors in mice [22]. Similarly Luo et al. described that chemoresitsant CD117^+^ cells isolated from xenografts displayed phenotypic feature of cancer stem cells such as serial transplantation and asymmetric division [29]. Recently, Gao et al. described that CD24^+^ population expressed increased level of some stem cells genes such as *Nestin*, **β*-catenin*, *Bmi-1*, *Oct4*, *Oct3/4*, *Notch1*, and *Notch4* compared to CD24^−^ and displayed quiescence, chemoresistance, and tumor initiation [20]. 

One of the challenges is to determine the hierarchy of the different markers described. In mammary gland, a hierarchy of stem cells is described using the different breast cancer stem cells markers [30]. Such hierarchy is essential to understand and identify the factors which regulate CSCs self-renewal versus proliferation and differentiation. Recently, Burgos-Ojeda et al. proposed a hierarchy for the OCSCs where they hypothesized that a common ovarian cancer stem cell can undergo asymmetric division to give rise to two different early OCSC progenitors ALDH^+^CD133^+^ (CD24^+/−^) or CD44^+^CD117^+^ (CD24^+/−^). Each of these early OCSC progenitors can then produce intermediate progenitor cells by asymmetric division which could produce more differentiated tumors [14].

The molecular drivers of the hierarchy can represent potentially important therapeutic targets. Several studies described correlations between OCSC markers and patient prognosis. Zhang et al. reported a poor prognosis associated with CD133 expression from a tumor bank of over 400 ovarian cancers [31]. More recently, Steg et al. showed the presence of CD133, ALDH1A1, and CD44 at low number in primary samples. The same markers were increased on a sample collected after chemotherapy and reduced back to initial level in recurrent tumor samples, suggesting their role in recurrence [32]. Using all these stem cells markers as target in a clinical trial seems to be the logical next step. Unfortunately, while limited numbers of tumors express CD133 (34–40%) or CD177 (30–40%), CD24 and CD44 are expressed in numerous tumors but targeting these cells *in vivo *is limited by wide expression of these molecules in normal tissues [22, 25, 33].

Stemness relies on a very precise equilibrium between the stem cells and the components of the niche. Recently, stem cells and their niches were identified in mammalian tissue such as the nervous system, muscle satellite cells, and spermatogonial stem cells [34, 35]. Many studies described how the niche and the stem cell interact through tissue-specific molecular signaling in maintaining stemness and inducing expansion of the stem cell population. For example, recent studies have clearly demonstrated the role of the endothelial niche in the expansion and maintenance of stemness of hematopoietic stem cells [36]. Molecular cues from stromal cells or the extracellular matrix will provide the signaling to maintain and expand the stem cell phenotype. 

The constitution of the tumor stroma brings another level of complexity. While for the sake of clarity we have separated different elements, most of them remain closely related and dependent.

## 3. Stromal Environment and Stemness

Cancer lesions are often perceived as never healing wounds with an inflammatory microenvironment. The infiltrating inflammatory cells include tumor promoting and tumor killing subtypes. Much molecular signaling can be hijacked by cancer cells and enhance tumorigenesis and progression toward a metastatic phenotype [37]. The stromal inflammatory reaction constitutes an environment containing many bioreactive molecules such as proliferative and survival signaling (EGF, FGF, HGF, IGF, or IL-6) that could enable CSCs maintenance and expansion. Moreover, many of these cytokines have been implicated in the occurrence of epithelial to mesenchymal transition (EMT) in many tumor types [38]. Recent lines of evidences have linked EMT phenotype to stemness [39, 40]. Therefore, we could assume that an inflammatory microenvironment will enable a subfraction of the tumor cells to gain/maintain a mesenchymal phenotype permissive to maintenance of stemness.

The tumor cells also participate to the inflammatory stroma as the upregulation of inflammatory molecules has been documented in the literature [11]. Ovarian cancers overexpress LL-37 (leucine, leucine 37) which is a member of the cathelicidin family of antimicrobial polypeptides. While LL-37 does not act directly on ovarian cancer cells, it attracts mesenchymal stem cells (MSCs) into ovarian tumor xenografts. MSC treated by LL-37 secreted increased amount of IL-1 receptor antagonist, IL-6, IL-10, CCL5, and VEGF and had a proangiogenic effect [41]. Long et al. demonstrated that CD133^+^ cells had increased expression of the chemokine CCL5 and its receptors, CCR1, CCR3, and CCR5, compared to CD133^−^ [42]. CCR5 mediated nuclear factor *κ*B (NF-*κ*B) dependent MMP9 secretion. These studies demonstrate the complex crosstalk relying on multiple cytokines and resulting in a permissive niche that will provide all molecular cues for maintenance and expansion of ovarian cancer stem cells.

The tumor stroma constitutes a hypoxic microenvironment before the appropriate signaling cues are able to induce neoangiogenesis. Hypoxia maintains and even upgrades stem cell characteristics [43, 44]. Under anaerobic conditions, glycolysis is favored and only a small amount of the pyruvate will be directed toward the mitochondria. The glycolytic metabolism activates tumor suppressor genes and oncogenes such as p53, RAS, or MYC. These oncogenes can activate HIF1*α* and HIF2 associated with activation of pluripotency marker genes such as *OCT4, SOX3*, and *KLF4* [45]. Liang et al. studied the effect of hypoxia on ovarian cancer stem cells [46]. They demonstrated increased ability for OCC to form spheres and colonies. CD44^bright^ displayed higher level of the stemness transcription factors OCT3/4 and Sox2 when cultured in hypoxic condition. In another study, CD44 and CD133 expression was increased through the Sox2 and OCT3/4 regulation in two different ovarian cancer cell lines (ES-2 and OVCAR3) [47].

## 4. Cellular Elements of the Stroma

The inflammatory stroma attracts other cell types such as mesenchymal stem cells and endothelial cells [48]. The protumoral and premetastatic roles of both cell types have been widely described in the literature. However few studies point out their interaction with cancer stem cells. There are many models of cellular interaction mediated by direct contact (tunneling nanotubes, synapses, trogocytosis) or microparticle mediation (Figures 1(c) to 1(f)). Their role in the maintenance of stemness remains to be clearly established. Mitsui et al. described increased expression of the CD133^+^ and sphere formation when Yolk sac ovarian cancer stem cells were cocultured with peritoneal MSCs [49]. The CD133^+^ cells displayed increased migration and invasion in culture with the MSCs. The crosstalk in this study seemed mediated through the SDF1/CXCR4 axis. In a more comprehensive approach, McLean et al. showed that cancer-associated MSCs (CA-MSC) had greater ability to increase tumor growth compared to normal MSCs [15]. They demonstrated that the CA-MSCs had abnormal BMP production. Treatment with recombinant BMP2 had the ability to increase ovarian cancer cell line stem cell population as defined by ALDH and CD133^+^ (up to 60%). Similarly the treatment of primary derived spheres with BMP2 also induced a 3.2- to 4.4-fold increase of the ALDH^+^ population.

As illustrated above, there is a strong interaction between cancer cells and the different elements of the niche. This crosstalk has a strong role in tumor biology as it participates to the plasticity of the tumor cells. Abelson et al. used the human embryonic stem cell derived experimental platform [50]. They isolated different clones from a single clear cell ovarian tumor. They showed that while some clones were not able to grow in a classical xenograft model their injection in a hESC-derived teratoma produced a tumor recapitulating the different cell populations of the primary tumor. More interestingly, they demonstrated that the microenvironment could switch the non-stem-cell EPCAM^+^CD44^−^ population to a stem cell EPCAM^+^CD44^+^ population. 

The microenvironment-dependent phenotypic plasticity has great therapeutic implication. This could result in failure of treatments targeting a single stable self-renewing clone. One option might then be to use multimodal approach to balance the equilibrium between the self-renewing and rapidly proliferating populations. Maintaining a permanent low level of self-renewing cell population will allow having a chronic disease rather than a rapidly lethal tumor. Many questions need to be answered such as whether self-renewal is a durable state rather than a dynamic niche dependent which is supported by many findings in the literature [51, 52]. This might also be dependent on tumor type as, for example, the Morrison groups demonstrated that nonputative stem cell population could give rise to a tumor in xenograft models when the microenvironment was modulated [53]. These findings were however obtained in the melanoma model and mice malignant peripheral nerve sheath tumors both originating from the neural crest. 

The role of endothelial cells in cancer stemness has not been yet clearly identified. Shank et al. have studied the action of metformin on ovarian cancer stem cells [54]. They demonstrated that metformin reduced ALDH^+^ CSC *in vitro* and *in vivo* and inhibited the growth of ovarian tumor spheres. One of the action of metformin resulted in decreased microvascular density consistent with previous studies [55, 56]. The data in the literature demonstrates that CSCs are highly angiogenic [25, 57] and that endothelial cells participating to neoangiogenesis provide essential growth factors for OCSCs [58].

## 5. Cytokines Environment and Global Crosstalk

Cytokines play an essential role in intercellular communications as described above. Many of them regulate stem cell phenotype in a variety of contexts ranging from normal development to neoplasia. Cao et al. showed that TGF*β* which is highly secreted in the ovarian cancer microenvironment induces tissue transglutaminase (TG2) expression and its enzymatic activity [59]. The treatment by TGF*β* induced spheroid formation enabling peritoneal dissemination. They demonstrated that TG2 was responsible for an EMT-mediated increase of the CD44^+^CD117^+^ population. Interestingly the effect of TGF*β* was greater when the ovarian cancer cells were cultured on fibronectin once again demonstrating the additive role of the different component of the microenvironment.

Among the different cytokines c-kit's role as a stem cell factor has already been described and plays a particular role. Indeed c-kit-mediated pathways are activated in cancer [60, 61]. Ovarian tumor abnormal expression of c-kit has been associated with poor prognosis. Chau et al. demonstrated increased expression of c-kit after enrichment for OCSC [62]. They showed that c-kit knockdown inhibited sphere formation. They displayed that hypoxia increased c-kit expression which in turn induced overexpression of the ABC drug ABCG2 transporter through the Wnt/*β*-catenin pathway, leading to chemoresistance in OCSC. One of the interesting findings of this group is that multiple rounds of chemotherapy seemed to enrich for OCSC harboring a high chemoresistance profile.

The crosstalk between cancer cells and the microenvironment has been illustrated in many other contexts. In a study by Ko et al., the authors showed a poor prognosis of tumors with HOXA9 expression [63]. *In vitro*, HOXA9 was not able to induce autonomous tumor cell growth. However ovarian cancer HOXA9 expression induced a cancer-associated fibroblasts phenotype for the peritoneal fibroblasts which stimulated OCC and endothelial cell growth. HOXA9 activated the transcription of TGF*β*2 which acted in a paracrine manner on peritoneal fibroblasts which in turn upregulated the protumoral panel of cytokines (CXCL12, IL-6, and VEGF-A) expression. This study illustrates the promotion of a permissive microenvironment which will provide the optimal ground for tumor growth. In accordance with these data, Alvero et al. demonstrated a very intricate relation between OCSC and the microenvironment [64]. They demonstrated that ovarian cancer stem cells participated to blood vessels and acquired markers of endothelial cell such as CD34 and VE cadherin. Interestingly, the process was not relying on VEGF but IKK*β*/NF*κ*b. While these data need to be confirmed by more functional studies demonstrating the ability of these cells to act as endothelial cells, the participation of the ovarian cancer stem cells to blood vessels beyond underlying their crosstalk with the endothelium could suggest resistance pathways to anti-VEGF-based targeted therapies.

## 6. Epithelial to Mesenchymal Transition (EMT)

Ovarian cancer represents a heterogeneous group of tumors with distinct clinical features, genetic alterations, and tumor behaviors. The phenomenon of EMT has been widely studied in ovarian cancer. The authors have suggested that OCC can undergo EMT to detach and MET to develop a metastatic nodule. These data should therefore be investigated in the context of OCSCs. Indeed targeting EMT has been suggested, as a potential treatment [65]. Several studies in breast cancer have demonstrated that EMT induced an increase in the CSCs population defined as CD44^+^CD24^−^ [66–68]. However, in 2012, Sarrio et al. indicated that a mesenchymal-like phenotype did not correlate with the acquisition of global stem cells/progenitors characteristics in breast cancer [69]. Supporting these findings Celià-Terrassa et al. showed that the acquisition of mesenchymal features (correlated with the loss of their epithelial properties) by cancer cells occurred at the expense of their self-renewal potential, in prostate and bladder cancer [70]. The body of data suggests that stemness might be a plastic phenotype that could depend both on tumor type and global stromal context.

In ovarian cancer, only a few studies have focused on the link between EMT and OCSCs. Recently, Jiang et al. demonstrated that the ovarian cancer cell lines displayed a side population with mesenchymal traits and typical mesenchymal genes. Inhibition of EMT process by Snail1 silencing decreased this side population occurrence and affected its invasive capacity and tumorigenicity *in vivo *[71]. Dahl Steffensen et al. established a correlation between the percentage of epithelial OCSCs and survival in early-stage ovarian cancer (FIGO I/II) in a cohort of 117 patients [72]. Concordant with findings in other epithelial cancers [70], Yin et al. demonstrated the ability of the OCSCs to generate peritoneal metastasis in an *in vivo* model. Furthermore they showed that TWIST-1 (a major transcription factor implicated in EMT) is constitutively degraded by the proteasome in OCSCs [73]. They suggest that OCSCs could be a source of ovarian cancer metastasis through balance of EMT/MET. 

## 7. Other Tumors

Ovarian cancer is not the only one to be maintained by a subpopulation of cells that display stem cell properties, mediate metastasis, and contribute to treatment resistance. A similar hierarchy governs many solids tumors, including breast [74], pancreas [75], glioblastoma [76], and prostate [77]. They are defined by different cell surface markers and characterized by specific phenotypic traits.

Several markers have been proposed in the literature to identify CSCs in many human cancers, but to date there is still no gold standard to define CSCs, leading to the hypothesis that the CSC phenotype might be dynamically switched [78]. Compared with the hematopoietic tumors, the properties of CSCs in solid tumors remained relatively undefined until recently. The first solid CSCs were identified in breast cancer by Al-Hajj et al. in 2003 with two surface markers CD44^+^/CD24^−/low^ [74]. Further characterization of breast CSCs was established using ALDH1 [79], mammosphere assay [80], and transplantation into immunodeficient mice [81]. For instance, CD133 has been described to be one of the most recurrent CSC markers in a number of solid malignancies, including brain tumor [82], prostate carcinoma [83], hepatocellular carcinoma [84], colorectal cancer [85, 86], and lung cancer [87].

All those CSCs are regulated by, and in turn regulate, cells within the tumor microenvironment. Recently, an emerging area of research supports that CSCs may promote tumor angiogenesis. As mentioned in glioma CSCs by Bao et al., the VEGF expression in CD133^+^ cells was 10–20-folds upregulated, combined with a significant increase in vascular density demonstrated by CD31 staining [57]. Furthermore, they described that therapy with VEGF antibody (bevacizumab) could reduce CSC-induced vascular endothelial cell migration and tube formation. They finally demonstrated, *in vivo*, that bevacizumab inhibited specifically the tumor growth of CSC-derived xenograft. Other studies support this finding that CSCs contribute to tumor vascular development in glioma [88, 89]. Cytokines produced by endothelial cells directly regulate CSCs contributing to their maintenance and their proliferation [90, 91]. This cross-regulation between CSCs and endothelial cells seems to be common in various solid tumor such as breast cancer [92], colon cancer [93], or brain tumor [94].

CSCs are also known to play a role in metastatic disease. Indeed, in breast cancer cells, CSCs can go through EMT via activation of Hedgehog (Hh), Wnt, Notch, or TGF*β* (transforming growth factor-*β*) leading to the upregulation of a group of transcriptional factors that drive EMT, resulting in the transformation of epithelial-like CSCs into cells with aggressive mesenchymal-like phenotypes [95]. All these pathways are induced by extracellular factors related to tumor microenvironment such as matrix metalloproteinase (MMP) family proteins [96]. The involvement of CSCs mesenchymal transition in metastatic spread was described in many tumors including head and neck squamous cell carcinoma [97], colorectal cancer [98], prostate cancer [99], and pancreatic cancer [100].

Inside their complex microenvironment, CSCs are also in close interaction with MSCs or tumor-associated macrophage [101]; however their precise interaction remains to be elucidated. 

## 8. Conclusion

**Figure 2:**
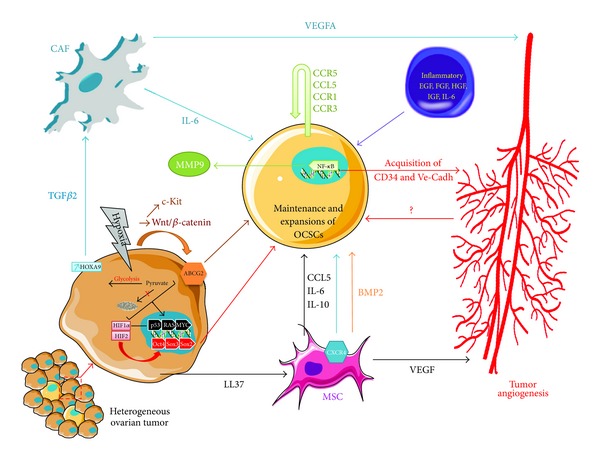
Maintenance and expansion of ovarian cancer stem cells by the tumor microenvironment. Schematic of the potential regulatory actors of the microenvironment in the maintenance of ovarian cancer stem cells.

The recent discovery of cancer stem cells in solid tumors mimicking leukemia has added another level of complexity to tumor heterogeneity. Indeed, the tumor now appears to be constituted by different cell populations harboring different phenotypes. Moreover, data presented above argue for a tremendous plasticity induced not only by clonal evolution but also by the interaction between the cancer cells and their microenvironment. It is difficult today to have a clear perception of the essential molecular hubs as the number of studies per disease is still limited and many studies have addressed few molecules rather than broad pathways (Figure 2). Accumulating evidence reveals that the composition of tumor microenvironments may define CSCs role throughout the different steps of carcinogenesis.

These findings have several consequences for patients' management. Indeed, as the tumor is now perceived as a dynamic structure, new factors (stem cell fraction, presence of stromal elements, and immune infiltrate) might be useful to predict prognosis. More importantly, we might have to consider absence of chemosensitivity rather than chemoresistance. Indeed, in the clonal theory of tumor evolution, upon treatment clones were able to develop resistance. In a more global approach, we could consider that at a time point some tumoral cells might not be sensitive to chemotherapy protected by their stemness and/or their interaction with the tumor microenvironment. Obviously, clonal and stem cell theories are not mutually exclusive, and under selective pressure the tumor plasticity could shift through clonal selection.

Therefore, there is a great need to gain a comprehensive understanding of the networks governing tumor plasticity, in particular the interaction between the stem cell compartment and the stroma. This will drive the design of new therapeutic approaches disrupting the tumor-stroma interaction to reduce tumoral plasticity.
